# Effects of intrarenal angiotensin 1–7 infusion on renal haemodynamic and excretory function in anaesthetised two‐kidney one‐clip and deoxycorticosterone acetate‐salt hypertensive rats

**DOI:** 10.1113/EP090791

**Published:** 2022-12-01

**Authors:** Elaine F. Barry, Mohammed H. Abdulla, Julie O'Neill, Sara AlMarabeh, Julie Beshara, Erin Parna‐Gile, Edward J. Johns

**Affiliations:** ^1^ Department of Physiology University College Cork Cork Republic of Ireland; ^2^ Department of Gastroenterology Mercy University Hospital Cork Republic of Ireland; ^3^ Department of Physiology University of Arizona Health Sciences Center Tucson AZ USA; ^4^ Department of Biopharmaceutics and Clinical Pharmacy School of Pharmacy University of Jordan Amman Jordan

**Keywords:** 2K1C, angiotensin (1–7), AT1 receptor, deoxycorticosterone acetate, Mas receptor

## Abstract

This study investigated the action of angiotensin 1–7 (Ang (1–7)) on renal haemodynamic and excretory function in the two‐kidney one‐clip (2K1C) and deoxycorticosterone acetate (DOCA)‐salt rat models of hypertension, in which the endogenous renin–angiotensin system (RAS) activity was likely to be raised or lowered, respectively. Rats were anaesthetised and prepared for the measurement of mean arterial pressure and kidney function during renal interstitial infusion of Ang (1–7) or saline. Kidney tissue concentrations of angiotensin II (Ang II) and Ang (1–7) were determined. Intrarenal infusion of Ang (1–7) into the clipped kidney of 2K1C rats increased urine flow (UV), absolute (U_Na_V) and fractional sodium (FE_Na_) excretions by 110%, 214% and 147%, respectively. Renal Ang II concentrations of the clipped kidney were increased with no major changes in Ang (1–7) concentration. By contrast, Ang (1–7) infusion decreased UV, U_Na_V, and FE_Na_ by 27%, 24% and 21%, respectively in the non‐clipped kidney in which tissue Ang (1–7) concentrations were increased, but renal Ang II concentrations were unchanged compared to sham animals. Ang (1–7) infusion in DOCA‐salt rats had minimal effects on glomerular filtration rate but significantly decreased UV, U_Na_V and FE_Na_ by ∼30%. Renal Ang (1–7) concentrations were higher and Ang II concentrations were lower in DOCA‐salt rats compared to sham rats. These findings demonstrate that the intrarenal infusion of exogenous Ang (1–7) elicits different renal excretory responses the magnitude of which is dependent on the balance between the endogenous renal Ang II–AT_1_ receptor axis and Ang (1–7)–Mas receptor axis.

## Abstract (highlights)

- **What is the central question of this study?**
  Are renal functional responses to intrarenal angiotensin 1–7 (Ang (1–7)) infusion dependent on the level of the endogenous renin–angiotensin system (RAS) in the two‐kidney one‐clip (2K1C) and deoxycorticosterone acetate (DOCA)‐salt animal models of hypertension?
- **What is the main finding and its importance?**
  The renal actions of Ang (1–7) are dependent on the relative endogenous levels of each arm of the classical angiotensin II–angiotensin II type 1 receptor (AT_1_R) axis and those of the Ang (1–7)–Mas receptor axis. These findings support the hypothesis that a balance exists between the intrarenal classical and novel arms of the RAS, and in particular the relative abundance of AT_1_R to Mas receptor, which may to a large extent determine the renal excretory response to Ang (1–7) infusion.

## 1 INTRODUCTION

Inappropriate activity of the renin–angiotensin system (RAS) is a major contributory factor in the development of cardiovascular and renal diseases through its fundamental role in regulating blood pressure and body fluid homeostasis (Yim & Yoo, 2008). The well‐studied pressor arm of the RAS, comprising angiotensin II (Ang II)–angiotensin‐converting enzyme (ACE)–angiotensin II type 1 receptors (AT_1_Rs), remains the central therapeutic target of these disease states through its blockade. Nonetheless, there is an increased prevalence of resistant hypertension among patients, and additional therapeutic options are required to reach target blood pressure ranges (Yaxley & Thambar, 2015). A depressor arm of the RAS, containing angiotensin‐converting enzyme 2 (ACE2)–angiotensin 1–7 (Ang (1–7))–Mas receptor, has been suggested as a protective pathway by opposing the molecular and cellular effects of Ang II (Padda et al., 2015; Patel et al., 2016). The potential of exploiting this novel RAS for the treatment of hypertension and other cardiovascular diseases deserves investigation (Padda et al., 2015).

In in vitro studies, Ang (1–7) presents as an intrarenal vasodilator and promotes natriuresis/diuresis via downregulation of sodium‐hydrogen exchanger‐3 (NHE‐3) in the renal proximal tubule (Dilauro & Burns, 2009; Padda et al., 2015; Santos et al., 2008). However, there is evidence from in vivo studies that the actions of Ang (1–7) are subject to change depending on endogenous conditions. Previous studies have suggested that Ang (1–7) acts as a counter‐regulatory mechanism in the classical RAS and that under conditions of reduced RAS activity, the actions of Ang (1–7) are blunted (O'Neill et al., 2013). Indeed, the study by O'Neill and others demonstrated that the Ang (1–7)‐induced diuresis and natriuresis were mediated by intrarenal Mas receptors (O'Neill et al., 2017), the magnitude of which was dependent on the dietary sodium content, Ang (1–7) increasing water and salt excretion following a low sodium diet and decreasing when there was a high sodium diet (O'Neill et al., 2017). These findings provided evidence that the ability of Ang (1–7) to increase fluid excretion was related to the degree of RAS activation. The work of Barry et al. (2021) also indicated that renal interstitial infusion of Ang (1–7) in normotensive control rats and spontaneously hypertensive rats significantly increased absolute sodium excretion (U_Na_V) in both groups but a greater degree in normotensive control rats despite both groups having a normal RAS (Barry et al., 2021). Considering these studies together, there has been a recognition that part of the variability in the reports of the renal vascular and excretory actions of Ang (1–7) may be dependent on not only different experimental conditions but also on the level of activation or suppression of the endogenous RAS and a ‘cross talk’ between the Mas and AT_1_ receptors. The hypothesis tested was whether the activation of the classical RAS in the two‐kidney one‐clip (2K1C) model would enhance renal functional responses to intrarenal Ang (1–7) infusion and whether there would be a depressing excretory response in the deoxycorticosterone acetate (DOCA)‐salt model due to suppression of the endogenous RAS.

## 2 METHODS

### 2.1 Ethical approval

Male Wistar rats of 100–125 g were sourced from Harlan (Bicester, UK) and delivered to University College Cork and kept in the Biological Services Unit in a 12:12 h light–dark cycle at a temperature of 21 ± 2°C with 50 ± 10% humidity. All animals had free access to standard rodent chow (Harlan‐Teklad, Bicester, UK) and tap water ad libitum. Animal studies were carried out in accordance with the European Union directive 2010/63/EU under project authorisations B100/3260 and B100/4481. Ethical approval was granted by the local Animal Experimentation Ethical Committee (AEEC) at University College Cork.

### 2.2 Surgical protocol in conscious rats

For the purposes of our studies, rats were distributed as groups of DOCA‐salt, sham DOCA, 2K1C and sham 2K1C rats. Twenty‐four hours prior to surgery, carprofen (Abbeyville Veterinary Hospital, Cork, Ireland) was given to rats in their drinking water (4 mg/kg/ml). Rats were anaesthetised using 5% gaseous isoflurane (Abbeyville Veterinary Hospital) at 500 ml/h O_2_ and maintained with 3% isoflurane at 200 ml/h O_2_. All animals were under observation for 2 h post‐surgery to ensure full recovery from anaesthesia. For 24 h after surgery, cages were placed on a heated pad and for 1 week post‐surgery, carprofen was added to the drinking water (4 mg/kg/ml). Animals were monitored daily post‐surgery for ripped stitches or any signs of wound infection, weight loss, dehydration or deviations from natural behaviour.

#### 2.2.1 2K1C Groups

Following anaesthesia, the left renal artery was exposed, isolated retroperitoneally and clipped with a silver clip (length 6 mm, width 1.5 mm, thickness 0.5 mm). Meanwhile, in sham 2K1C rats, the left renal artery was exposed and isolated without clipping. Thereafter, the muscle and skin layers were cleaned and sutured, and the animal was allowed to recover. Renal functional studies were commenced 3 weeks post‐surgery. Blood pressure was assessed at this time mark through femoral artery cannulation as described in the surgical protocol below and only animals with a mean arterial pressure higher than 140 mmHg were included in studies of the 2K1C hypertensive group.

#### 2.2.2 DOCA salt and sham DOCA

In DOCA‐salt and sham DOCA rats, the right kidney was exposed retroperitoneally and a sterile prolene polypropylene suture (Ethicon, Johnson & Johnson Intl, Scotland, UK) was used to tie off the right renal artery, vein and ureter, followed by a right nephrectomy. One week post‐surgery, DOCA rats were provided with ad libitum drinking water that contained 0.9% NaCl and 0.2% KCl. DOCA‐treated animals are subject to mild hypokalaemia (Ueno et al., 1988) and therefore they were provided with 0.2% KCl in the drinking water to offset electrolyte imbalance (Sahin‐Erdemli et al., 1995). In addition, these rats were given subcutaneous injections of 15 mg/kg DOCA (Sigma‐Aldrich Ltd, Arklow, Ireland) every 3 days for a further 4 weeks. DOCA salt was prepared by suspending 15 mg of DOCA in 2 ml arachis oil. For sham DOCA rats, ad libitum tap drinking water was provided along with subcutaneous injections of 2 ml/kg arachis oil every 3 days for a further 4 weeks.

### 2.3 Terminal studies

Animals were anaesthetised using 60 mg/kg sodium pentobarbital (Euthatal, Merial Animal Health Ltd, Berkshire, UK). The trachea was cannulated with PE240 tubing (ID 1.86 mm, Smiths medical, Ashford, UK) to maintain airway patency. An arterial cannula (PE25, ID 0.40 mm, Smiths) filled with heparinised saline (20 U/ml) was connected to a blood pressure transducer (MLT844, ADInstuments Ltd, Oxford, UK) that was linked to a PowerLab system (ADInstuments) to record arterial blood pressure. The same femoral arterial cannula was used to collect blood samples during the experiment. A PE50 (ID 0.58 mm) cannula was inserted into the femoral vein for continuous infusion of saline (Perfusor Space Infusion Pump System, B. Braun, Bethlehem, PA, USA) throughout the experimental protocol at an infusion rate of 3 ml/h. Supplementary anaesthesia was provided through the femoral venous line at regular intervals during the experiment. The ureter was cannulated with PE10 (ID 0.28 mm, Smiths) for the collection of urine. A saline‐filled PE10 cannula was inserted into the cortex of the kidney to allow intra‐renal infusion of either saline or Ang 1–7 (3 × 10^−3^ M, Sigma‐Aldrich) at an infusion rate of 1 ml/h using a Hamilton syringe pump (KD Scientific, Holliston, MA, USA). Intrarenal infusions were carried out into the left kidneys of all groups. In an additional group of 2K1C rats, intrarenal infusions were preformed into the right kidney to examine renal function in the non‐clipped kidneys. Following surgical instrumentation, a 2 ml bolus of fluorescein isothiocyanate (FITC)‐inulin saline was injected intravenously followed by a continuous intravenous infusion of FITC‐inulin at a rate of 10 mg/kg/h throughout the study to allow measurement of glomerular filtration rate (GFR). The animal was allowed a recovery period of 1.5 h from the surgical procedures to ensure stabilization of blood pressure and urine flow before commencing the following experimental protocol.

### 2.4 Experimental protocol of terminal experiments

The 2K1C acute studies comprised three groups of rats:

- Sham 2K1C group (*n* = 7): saline or Ang (1–7) was infused into the left kidney in this group of rats.
- Clipped kidney‐2K1C (CK‐2K1C) group (*n* = 8): saline or Ang (1–7) was infused into the left clipped kidney.
- Non‐clipped kidney‐2K1C (NCK‐2K1C) group (*n* = 7): saline or Ang (1–7) was infused into the right non‐clipped kidney.

The DOCA acute studies on the other hand comprised two groups of rats:

- Sham DOCA group (*n* = 6): saline or Ang (1–7) was infused into the left kidney in this group of rats.
- DOCA‐salt group (*n* = 7): saline or Ang (1–7) was infused into the left kidney in this group of rats.

**FIGURE 1:**
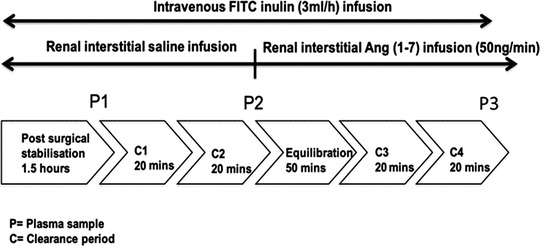
A schematic representation of the experimental protocol. In 2K1C, DOCA‐salt‐treated and corresponding sham rats, following a 1.5 h stabilization period, an arterial blood sample was withdrawn. After intra‐renal infusion of saline, two urine samples were collected, each of 20 min, which was then followed by the withdrawal of a second blood sample. This was then followed by a 50‐min equilibration period during which Ang (1–7) was infused into the kidney instead of saline. Two further urine samples were collected, each of 20 min. Then, a third blood sample was withdrawn. P, plasma sample; C, clearance (urine sample)

The full protocol is shown in Figure 1. Following a stabilisation period, a blood sample (400 μl) was withdrawn from the femoral arterial cannula, centrifuged and plasma was extracted. An equivalent volume of heparinized saline was added to the remaining red blood cells, which were re‐infused through the arterial cannula. Two 20 min baseline urine samples were collected during the intrarenal infusion of saline after which the intrarenal infusion of saline was switched to Ang (1–7) (3 × 10^−3^ M) for 50 min. A further blood sample was withdrawn, and two 20 min urine samples were collected. After the collection of urine samples, a third blood sample was withdrawn (Figure 1). Animals were then killed with an overdose of sodium pentobarbital. Urine and plasma samples were analysed immediately after killing.

### 2.5 Renal function analysis

Renal functional analysis involved the measurement of GFR, urine flow (UV), absolute sodium excretion (U_Na_V), clearance of sodium (Cl_Na_) and fractional sodium excretion (FE_Na_). Sodium content in plasma and urine was analysed using flame photometry (Sherwood, M410, Sherwood Scientific Ltd, Cambridge, UK). U_Na_V was calculated as: U_Na_V = UV × U_Na_/1000, where UV is urine volume and U_Na_ is the concentration of sodium in the urine. Clearance of sodium was calculated as: Cl_Na_ = UV × U_Na_/P_Na_ × *T* × BW, where P_Na_ is the plasma concentration of sodium, *T* is duration of urine collection and BW is body weight. FE_Na_ was calculated as: FE_Na_ = Cl_Na_ × 100/GFR.

In acute renal functional experiments, inulin concentration in urine and plasma samples was analysed using a fluorometric multilabel counter plate reader set at a wavelength of 520 nm (Victor 2, Perkin Elmer, Waltham, MA, USA) and processed using Wallac Workstation software (Perkin Elmer). Inulin measurements were used to calculate GFR as follows: GFR = (UV × U_inulin_)/(P_inulin_ × *T* × BW). The inulin concentration values overtime periods of 20 min were used for the calculation of acute GFR responses. An average of the first two urine samples for each parameter during the intrarenal saline infusion served as baseline renal function and an average of the third and fourth urine samples for each parameter were calculated during the intrarenal Ang (1–7) infusion.

### 2.6 Enzyme‐linked immunosorbent assay

The renal cortical and medullary concentrations of Ang II and Ang (1−7) were determined as previously described (Barry et al., 2021). Briefly, the kidney was separated into cortex and medulla and the tissues were then homogenised followed by centrifugation for 5 min at 4°C and the resulting supernatant was stored at −80°C for further analysis. During the assay, the samples were added to enzyme‐linked immunosorbent assay (ELISA) assay wells that were coated with either primary anti‐Ang (1−7) or anti‐Ang II antibody in duplicates. The Ang II and Ang (1–7) ELISA kits were sourced from Cusabio Biotech Co. Ltd, Wuhan, China. Further steps in this assay included incubation at 37°C for 2 h to facilitate the binding of the protein to the antibody. This was followed by another step that required incubation for 1 h to allow binding between the protein of interest and the detection antibody. Once all steps of the assay were completed, the optical density of each well was measured, as per the manufacturer's instructions, using a SpectraMax M3 microplate reader and processed by Softmax Pro 6 software (Molecular Devices, San Jose, CA, USA). The readings from the plate reader were finally utilised to determine the tissue concentrations of Ang II and Ang (1–7).

### 2.7 Tissue homogenisation

The renal tissue for western blot analysis was homogenised using a 10 mM Tris/250 mM sucrose buffer (pH 7.4). Approximately, 0.25 g of tissue was thawed on ice and a homogenisation buffer was added at a ratio of 100 mg tissue: 1000 μl buffer. Each sample was homogenised on ice using an Omni International (Kennesaw, GA, USA) tissue homogeniser. Supernatants were snap‐frozen and stored at −80°C. The renal tissue for ELISA was homogenised using phosphate buffered saline of pH 7.1 instead of Tris/sucrose buffer. Protein content in each homogenised sample was determined using a Pierce bicinchoninic acid assay kit (Thermo Fisher Scientific, Waltham, MA, USA), as per the manufacturer's instructions

### 2.8 Western blotting

Renal tissue protein (30 μg) was loaded in each well of a 5% stacking gel. Samples were separated on a 10% resolving gel as previously described (Barry et al., 2021). Proteins were separated by molecular mass using SDS‐PAGE. A Trans‐Blot SD transfer cell (Bio‐Rad Laboratories, Hercules, CA, USA) was used for the electrophoretic transfer, and the success of the transfer was evaluated using reversible Ponceau S staining. The membrane was washed in 5% non‐fat dried milk made up with 1× Tris‐buffered saline–Tween (TBST) for an hour to block the surface of the membrane. Then, anti‐AT_1_R antibody (1:2000, Alpha Diagnostics, San Antonio, TX, USA) was diluted in 5% non‐fat milk in 1× TBST, and the membrane was incubated overnight with the primary antibody at 4°C. The membrane was then washed four times, each of 7 min, in 1× TBST with gentle agitation to remove any unbound primary antibody. Anti‐rabbit IgG peroxidase conjugate (Sigma‐Aldrich) was diluted to 1:2000 in the blocking solution and it was incubated with the washed membrane using gentle agitation at room temperature for 1 h. Bands were visualised using enhanced chemiluminescence (Sigma‐Aldrich). The excess enhanced chemiluminescence was blotted off using absorbent tissue and the membrane was placed face down on the LI‐COR C‐DIGit Blot Scanner (LI‐COR Biotechnology, Cambridge, UK). The digital images from the scanner were obtained with Image Studio software (LI‐COR Biotechnology). Image J software (National Institutes of Health, Bethesda, MD, USA) was used to measure the amount of total protein and protein of interest in each sample. The expression of the protein of interest was normalised to total protein (Ponceau S loading control), which was measured by densitometry.

### 2.9 Statistical analysis

Data were analysed using Prism 6 software (GraphPad Software, Inc., San Diego, CA, USA) and are presented as means ± SD. Body and kidney weights were compared between groups using a Mann–Whitney test. The mean arterial pressure (MAP) and renal functional parameters obtained during the intra‐renal infusion of Ang (1–7) were compared with those during the intra‐renal infusion of saline using two‐way ANOVA or two‐way repeated measure ANOVA as appropriate followed by the Holm–Šidák multiple comparison test. The renal cortical and medullary content of Ang II and Ang (1–7) were compared between different groups using the Mann–Whitney test. AT_1_ and Mas receptor protein expression was compared between groups using the Mann–Whitney test or Kruskal–Wallis test followed by Dunn's multiple comparisons test where appropriate. Statistical significance was taken at *P* < 0.05.

## 3 RESULTS

### 3.1 Baseline data

#### 3.1.1 2K1C model

Baseline MAP was significantly (*P* < 0.0001) higher in the 2K1C group, 181 ± 21 mmHg, compared to the sham group, 116 ± 7 mmHg, but the body weights of the 2K1C and sham groups were similar (245 ± 25 vs. 259 ± 20 g, *P* = 0.096). Meanwhile, the left renal mass of the clipped kidney (percentage of body weight) of the 2K1C rats was significantly lower (*P* = 0.02) compared to the sham rats (0.35 ± 0.05 vs. 0.39 ± 0.05%). However, in the 2K1C hypertensive rats the contralateral (right) non‐clipped kidney showed evidence of hypertrophy (*P* < 0.0001) when compared to the clipped kidney of the same rat (0.52 ± 0.11 vs. 0.35 ± 0.05%) or with the right kidney of the sham rats (0.52 ± 0.11 vs. 0.39 ± 0.05%).

#### 3.1.2 DOCA model

MAP was significantly higher (*P* = 0.002) in DOCA‐salt‐treated rats after 4 weeks of DOCA‐salt treatment, at 158 ± 16 mmHg compared to sham, at 107 ± 19 mmHg. There were no significant differences in body weight between the DOCA‐salt and the respective sham group (309 ± 30 vs. 332 ± 29 g, *P* = 0.059). In the DOCA model, the weight of the remaining left kidney of each rat was normalised to body weight and was significantly higher (*P* = 0.0002), by ∼48%, than the left kidney mass of sham rats of this model (0.90 ± 0.17 vs. 0.61 ± 0.08%).

### 3.2 Renal excretory function

#### 3.2.1 2K1C model

**FIGURE 2:**
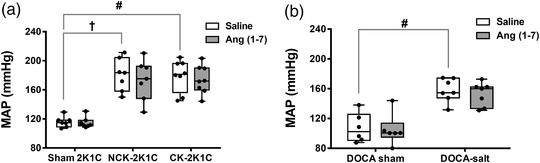
Baseline MAP during the intrarenal infusion of saline and Ang (1–7). (a, b) Box and whiskers plots for each group, where each point represents MAP obtained from a single rat. MAP was not altered during the intrarenal infusion of Ang (1–7) compared to the intrarenal infusion of saline into the left kidney in any of the groups of 2K1C (sham, *n* = 7; NCK, *n* = 7; CK, *n* = 8) and DOCA (sham, *n* = 6; DOCA‐salt, *n* = 7) models. MAP remained relatively stable during the intrarenal infusion of Ang (1–7) compared to baseline in all groups of 2K1C and DOCA models. #*P* < 0.05 CK‐2K1C or DOCA‐salt versus sham 2K1C or sham DOCA, respectively; †*P* < 0.05 NCK‐2K1C versus Sham 2K1C. 2K1C; two‐kidney one‐clip; Ang (1–7), angiotensin (1–7); CK, clipped kidney; DOCA, deoxycorticosterone acetate; MAP, mean arterial blood pressure; NCK, non‐clipped kidney

The acute renal functional studies comprised three groups of rats: a sham 2K1C group, in which saline or Ang (1–7) was infused into the left kidney; a 2K1C group in which saline or Ang (1–7) was infused into the left clipped kidney (CK‐2K1C); and a 2K1C group in which saline or Ang (1–7) was infused into the right non‐clipped kidney (NCK‐2K1C). The intrarenal infusion of saline or Ang (1–7) did not change blood pressure in any of the sham, CK‐2K1C or NCK‐2K1C groups (Figure 2a).

**FIGURE 3:**
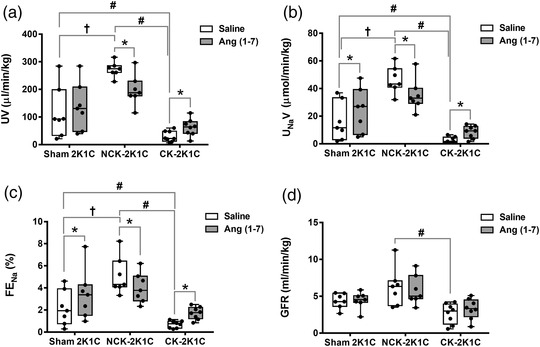
The effect of intrarenal Ang (1–7) on kidney function in 2K1C rats. Each panel shows box and whisker plot of each renal functional parameter, where each point represents kidney function obtained from a single rat. Figure shows UV (a), U_Na_V (b), FE_Na_ (c) and GFR (d) obtained from sham 2K1C rats (*n* = 7), from the non‐clipped kidney (*n* = 7) and from the clipped kidney (*n* = 8) of 2K1C rats. *P < 0.05 Ang (1–7) versus saline, #*P* < 0.05 CK‐2K1C versus NCK‐2K1C or Sham 2K1C, †*P* < 0.05 NCK‐2K1C versus sham 2K1C. 2K1C; two‐kidney one‐clip; CK, clipped kidney; FE_Na_, fractional sodium excretion; GFR, glomerular filtration rate; NCK, non‐clipped kidney; U_Na_V, absolute sodium excretion; UV, urine flow rate

Baseline UV in the CK‐2K1C group was significantly lower while GFR was not changed during the intrarenal infusion of saline compared to the sham 2K1C group (UV: 29.7 ± 19.8 vs. 116.1 ± 93.6 μl/min/kg, *P* = 0.007; GFR: 2.66 ± 1.37 vs. 4.38 ± 2.60 ml/min/kg, *P* = 0.078). By contrast, the baseline UV in the sham 2K1C group was significantly smaller compared to the NCK‐2K1C group (*P* < 0.0001, Figure 3a). UV and GFR in the CK‐2K1C group were significantly lower (UV: *P* < 0.0001; GFR: *P* = 0.0005, Figure 3a,d) compared to the NCK‐2K1C group.

During saline infusion, U_Na_V was significantly lower in the CK‐2K1C group than in the left kidney of sham 2K1C rats (*P* = 0.019) and the NCK‐2K1C rats (2.72 ± 2.17 vs. 16.08 ± 13.92 vs. 46.37 ± 9.74 μmol/min/kg, *P* < 0.0001), but was significantly greater in the NCK‐2K1C rats compared to sham 2K1C rats (*P* < 0.0001; Figure 3b). Moreover, FE_Na_ over the period of saline infusion was significantly higher in the NCK‐2K1C rats compared to the CK‐2K1C (*P* < 0.0001) and sham 2K1C rats (*P* = 0.0009) (5.11 ± 1.69% vs. 0.69 ± 0.33% vs. 2.19 ± 1.59%; Figure 3c).

The intrarenal infusion of Ang (1–7) increased UV in the left kidney of the CK‐2K1C group by 110% (*P* = 0.043), whereas it was decreased by 27% in the NCK‐2K1C group (*P* = 0.0001), compared to the intrarenal infusion of saline (Figure 3a). Ang (1–7) infusion increased U_Na_V and FE_Na_ by 51% (*P* = 0.019) and 57% (*P* = 0.001), respectively in the sham 2K1C group and by 214% (*P* = 0.041) and 147% (*P* = 0.002), respectively, in the CK‐2K1C group but decreased by 24% (*P* = 0.003) and 21% (*P* = 0.002), respectively, in the NC‐2K1C group (Figure 3b,c). GFR was unchanged by the intrarenal infusion of Ang (1–7) compared to the intrarenal infusion of saline in any of the groups (Figure 3d).

#### 3.2.2 DOCA model

**FIGURE 4:**
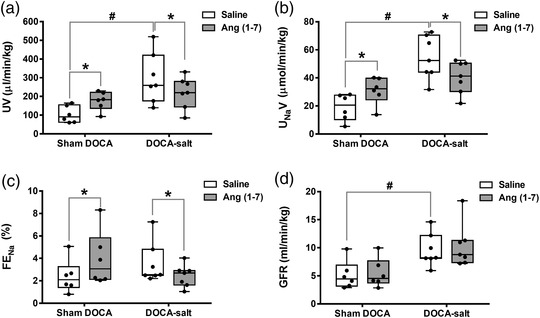
The effect of intrarenal Ang (1–7) on kidney function in sham DOCA and DOCA‐salt‐treated rats. Each panel shows box and whisker plot of each renal functional parameter, where each point represents renal excretory parameter obtained from a single rat. Figure shows UV (a), U_Na_V (b), FE_Na_ (c) and GFR (d) obtained from sham DOCA rats (*n* = 6) and DOCA‐salt‐treated rats (*n* = 7) during the intra‐renal infusion of Ang (1–7) and saline. *P < 0.05 Ang (1–7) versus saline, #*P* < 0.05 DOCA‐salt versus sham DOCA. DOCA, deoxycorticosterone acetate; FE_Na_, fractional sodium excretion; GFR, glomerular filtration rate; U_Na_V, absolute sodium excretion; UV, urine flow rate

Intrarenal infusion of Ang (1–7) did not change MAP in DOCA‐salt‐treated or sham DOCA rats and was comparable to that during intrarenal saline infusion (Figure 2b). Basal UV (*P* = 0.001), U_Na_V (*P* < 0.0001) and GFR (*P* = 0.019) were significantly higher in the DOCA‐salt‐treated group compared to the sham DOCA group (UV: 298 ± 134 vs. 104 ± 45 μl/min/kg; U_Na_V: 54.3 ± 15.4 vs. 19.0 ± 9.5 μmol/min/kg; GFR: 9.60 ± 2.93 vs. 5.13 ± 2.53 μl/min/kg; Figure 4a,b,d). However, basal FE_Na_ was not significantly different between groups (2.39 ± 1.47% vs. 3.58 ± 1.85%; Figure 4c).

Intrarenal infusion of Ang (1–7) increased UV by 70% (*P* = 0.037) in sham DOCA rats compared to the intrarenal infusion of saline (Figure 4a). Conversely, Ang (1–7) resulted in a decrease (*P* = 0.037) in UV by ∼26% in the DOCA‐salt‐treated rats (Figure 4a). However, the intrarenal infusion of Ang (1–7) was not associated with any changes in GFR compared to the intrarenal infusion of saline in DOCA‐salt‐treated and sham DOCA rats (Figure 4d).

The intrarenal infusion of Ang (1–7) significantly (*P* = 0.013) increased U_Na_V (by ∼63%) and FE_Na_ (by ∼65%) in the sham DOCA group compared to the intrarenal infusion of saline (Figure 4b,c). By contrast, in DOCA‐salt‐treated rats, the intrarenal infusion of Ang (1–7) significantly decreased U_Na_V, by ∼26% (*P* = 0.007), and FE_Na_, by ∼32% (*P* = 0.023), compared to the intrarenal infusion of saline (Figure 4b,c).

#### 3.2.3 Renal Ang II and Ang (1–7) concentrations

**FIGURE 5:**
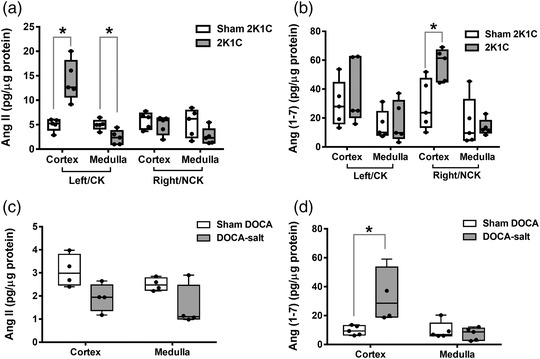
Tissue concentration of Ang II and Ang (1–7). (a, b) Concentration of Ang II and Ang (1–7) in the kidneys of sham 2K1C (*n* = 5) and 2K1C rats (*n* = 5). (c, d) Concentration of Ang II (sham DOCA, *n* = 4; DOCA‐salt, *n* = 4) and Ang (1–7) (sham DOCA, *n* = 5; DOCA‐salt, *n* = 5) in the kidneys of sham DOCA and DOCA‐salt‐treated rats. (a, b) *P < 0.05 2K1C versus sham 2K1C; (c, d) *P < 0.05 DOCA‐salt versus sham DOCA. 2K1C; two‐kidney one‐clip; Ang II, angiotensin II; Ang (1–7), angiotensin 1–7; CK, clipped kidney; DOCA, deoxycorticosterone acetate; NCK, non‐clipped kidney

Tissue levels of Ang II and Ang (1–7) are shown in Figure 5. Ang II concentrations in the cortex of the clipped kidney of 2K1C rats were higher than Ang II concentrations in the same kidney of sham 2K1C rats (*P* = 0.008, Figure 5a). However, Ang II levels in the medulla of the clipped kidney of 2K1C rats were lower than Ang II levels in the medulla of the same kidney in sham 2K1C rats (*P* = 0.016). Ang (1–7) levels were elevated in the renal cortex of the non‐clipped kidney of 2K1C rats compared to Ang (1–7) concentrations in the right renal cortex of sham 2K1C rats (*P* = 0.032).

Ang II concentrations in the renal cortex of DOCA‐salt‐treated rats were lower than in the renal cortex of sham DOCA rats (*P* = 0.057, Figure 5c). Conversely, Ang (1–7) concentrations in the renal cortex of DOCA‐salt‐treated rats were significantly higher than in the renal cortex of sham DOCA rats (*P* = 0.016, Figure 5d).

### 3.3 Protein expression of Ang II and Ang (1–7) receptors

#### 3.3.1 AT_1_ receptor

**FIGURE 6:**
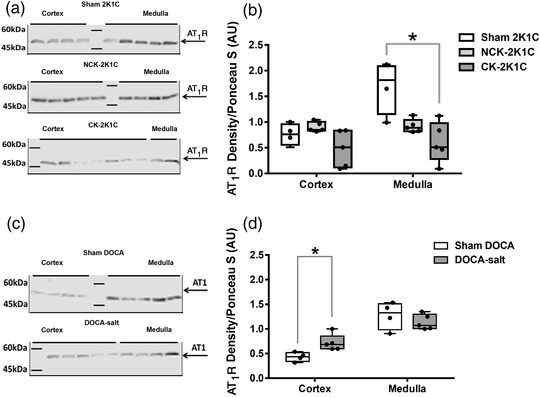
Protein expression of AT_1_ receptor in the cortex and medulla of 2K1C and DOCA rats. (a, c) Representative western blots with a band around 52 kDa marking the protein expression of AT_1_R in the cortex and medulla of the sham 2K1C (*n* = 4), NCK‐2K1C (*n* = 5) and CK‐2K1C rats (*n* = 5). (b) Relative AT_1_R density (normalised to Ponceau S loading control) in the cortex and medulla of sham 2K1C and 2K1C groups. (d) Relative AT_1_R density in the cortex and medulla of DOCA‐salt‐treated (*n* = 4) and sham DOCA (*n* = 5) rats. *P < 0.05 versus sham in 2K1C or DOCA groups. 2K1C; two‐kidney one‐clip; AT_1_R, angiotensin II type 1 receptor; CK, clipped kidney; DOCA, deoxycorticosterone acetate; NCK, non‐clipped kidney

Representative western blots for AT_1_R protein expression in the renal cortex and medulla of 2K1C and DOCA groups are shown in Figures 6a,c. AT_1_R expression showed a 44% (*P* = 0.070) attenuation in the renal cortex of the CK‐2K1C but was not statistically significantly different from AT_1_R expression in the renal cortex of sham 2K1C rats (Figure 6b). There was no significant difference in AT_1_R expression between the renal medulla of the NCK‐2K1C group and renal medulla of the sham 2K1C group. However, AT_1_R expression in the renal medulla of CK‐2K1C group was significantly (*P* = 0.020) lower than in the renal medulla of the sham 2K1C group (Figure 6b).

Protein expression of AT_1_Rs in the renal cortex of DOCA‐salt‐treated rats was 67% greater (*P* = 0.016), compared to that in the renal cortex of sham DOCA rats (Figure 6d). There was no significant difference in the expression of AT_1_Rs in the renal medulla between groups (Figure 6d).

#### 3.3.2 Mas receptor

**FIGURE 7:**
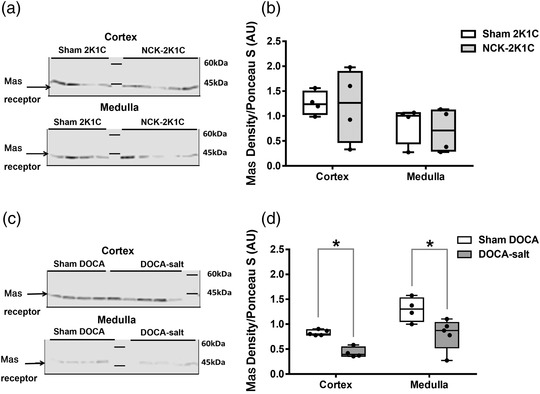
Protein expression of Mas receptor in the renal cortex and medulla of 2K1C and DOCA rats. (a, c) Representative western blots with a band around 45 kDa marking the protein expression of Mas receptor in the cortex and medulla of the sham 2K1C (*n* = 4) and NCK‐2K1C (*n* = 4) rats. (b) Relative Mas receptor density (normalised to Ponceau S loading control) in the cortex and medulla of sham 2K1C and NCK‐2K1C groups. (d) Relative Mas receptor density in the cortex and medulla of DOCA‐salt‐treated (*n* = 4) and sham DOCA (*n* = 5) rats. *P < 0.05 DOCA‐salt versus sham DOCA. 2K1C; two‐kidney one‐clip; DOCA, deoxycorticosterone acetate; NCK, non‐clipped kidney

Representative western blots of the Mas receptor protein expression in the renal cortex and medulla of 2K1C and DOCA groups are shown in Figures 7a,c. There was no significant difference in the expression of Mas receptor in the renal cortex or medulla of the NCK‐2K1C group compared to sham 2K1C rats (Figure 7b). However, the relative protein expression of Mas receptor was significantly lower in the renal cortex (∼48%, *P* = 0.016) and renal medulla (∼38%, *P* = 0.032) of DOCA‐salt‐treated rats compared to sham DOCA rats (Figure 7d).

## 4 DISCUSSION

In this study, we aimed to evaluate renal haemodynamic and excretory functional responses to exogenous intrarenal Ang (1–7) in two rat models of hypertension, one in which activity of the renin–angiotensin system would be elevated, the 2K1C model, and one in which it would be depressed, the DOCA‐salt model. Changes in the intra‐renal concentrations of Ang II and Ang (1–7), and renal cortical and medullary AT_1_ and Mas receptor protein expression were measured in an attempt to reveal potential underlying mechanisms. The intrarenal infusion of Ang (1–7) into the clipped kidney of 2K1C rats, in which Ang II, but not Ang (1–7), concentrations were markedly elevated, caused a natriuresis and diuresis, the magnitude of which was significantly larger than that obtained in the corresponding sham group of rats. By contrast, the intrarenal infusion of Ang (1–7) into the non‐clipped kidney in 2K1C rats, in which renal tissue Ang II concentrations were unchanged but Ang (1–7) levels were elevated compared to sham rats, caused a small anti‐diuresis and anti‐natriuresis. These findings provide further evidence for a relationship between renal cortical levels of Ang II and the excretory responses to Ang (1–7). Intrarenal infusion of Ang (1–7) in the sham DOCA rats caused a diuresis and natriuresis but in the DOCA‐salt‐treated group, in which renal cortical Ang II concentrations were depressed but Ang (1–7) levels were increased, caused an anti‐diuresis and anti‐natriuresis. Whether the renal functional responses to Ang (1–7) are modified during hypertension in the 2K1C and DOCA‐salt models remains to be determined, but previous findings using the spontaneously hypertensive rat model suggested that hypertension per se is not a factor in determining the renal responses to Ang (1–7) in this model (Barry et al., 2021).

The 2K1C model is an experimental rat model of human renovascular hypertension, where hypertension development, at least in the early phases, is dependent on increased circulating levels of Ang II, originating from the clipped kidney. The placement of a clip on the left renal artery resulted in a lower renal mass of the clipped kidney and hypertrophy of the contralateral non‐clipped kidney compared to the kidneys of sham 2K1C rats. Baseline levels of urine flow, and absolute and fractional sodium excretions in the present study were much lower in the clipped compared to the non‐clipped kidneys. This differential has been reported previously (Kopp & Buckley‐Bleiler, 1989) and was most likely due to a number of factors including the initial reduction in perfusion pressure distal to the clip initiating an activation of the intrarenal RAS (Guan et al., 1992; Prieto‐Carrasquero et al., 2008). The acute intrarenal infusion of Ang (1–7) elicited a marked diuresis and natriuresis from the clipped kidney of 2K1C rats and in the sham group without change in GFR. The magnitude of the excretory responses was proportionately larger in the clipped kidney of the 2K1C compared to the sham group. Previous studies have shown that Ang (1–7) elicited a diuretic and natriuretic response (DelliPizzi et al., 1994; Heller et al., 2000; Vallon et al., 1997), the magnitude of which was greater in rats with an enhanced RAS induced by feeding a sodium‐restricted diet (O'Neill et al., 2013). Indeed, renal cortical Ang II concentrations in the clipped kidneys of the 2K1C rats were some threefold higher compared to the cortical levels in the sham group of animals. Conversely, intrarenal infusion of Ang (1–7) into the non‐clipped kidney of 2K1C rats resulted in an anti‐diuretic and anti‐natriuretic effect even though renal cortical Ang II concentrations were no different from those of the sham rats. The underlying reason for this differential action of Ang (1–7) on renal excretory responses may reside not only in the renal concentrations of Ang II but also of Ang (1–7), which were markedly elevated in the non‐clipped kidney.

The DOCA‐salt rat is a well‐defined model of experimental hypertension. Baseline MAP, water turnover and U_Na_V were all significantly higher in DOCA‐salt‐treated rats compared to the sham DOCA rats, as has been reported previously (Jacob et al., 2005). Interestingly, intrarenal infusion of Ang (1–7) in the DOCA‐salt‐treated rats caused small reductions in UV, U_Na_V and FE_Na_ which contrasted to the approximate doubling of these variables in the sham DOCA rats. An anti‐diuretic response to peripherally administered Ang (1–7) has been reported previously in water loaded rats (Santos & Baracho, 1992) and aligns with a previous study which reported a blunted diuretic and natriuretic response to intrarenal Ang (1–7) infusion in rats fed a high sodium diet with a suppressed RAS (O'Neill et al., 2013). The reason for this blunted response to Ang (1–7) is unclear but may reflect an interaction between Ang II and Ang (1–7) and their receptors. Indeed, Ang (1–7) has been previously proposed to antagonise the renal effects of Ang II, with Ang (1–7) producing a natriuresis and diuresis and Ang II causing an anti‐diuresis and anti‐natriuresis (Chappell et al., 1998). On the other hand, in an in vitro study by Xue et al. (2012), both Ang (1−7) and Ang II had similar effects on cell proliferation, phosphorylation and TGF‐β1 and extracellular matrix synthesis in cultured renal mesangial cells. It is important to note that the anti‐diuretic and anti‐natriuretic responses to Ang (1–7) in the non‐clipped kidney and DOCA‐salt rats were associated with either an unchanged or depressed renal Ang II but elevated Ang (1–7) content. By contrast, the diuretic and natriuretic responses to exogenous Ang (1–7) in the sham rats occurred against a normal background renal concentration of Ang II and Ang (1–7).

To establish what factors may affect renal functional responses to Ang (1–7), baseline MAP, renal functional parameters and the RAS peptides were measured. Angiotensin II concentrations in the cortex of the clipped kidney were greatly increased 25 days after clipping, which was consistent with previous reports in this time frame (Guan et al., 1992; Prieto‐Carrasquero et al., 2008). Interestingly, Ang II levels in the medulla of the clipped kidney were lower than in the left medulla of sham 2K1C rats, which contrasts with the report of Prieto‐Carrasquero et al. (2008) that Ang II levels were significantly higher in the medulla of the clipped kidney of 2K1C rats compared to the renal medulla of sham rats. However, those authors used a different experimental scenario in which blood was obtained from conscious rats following decapitation as against anaesthetised surgically stressed rats in the current study. Indeed, plasma and renal tissue concentrations of Ang II were reported to be higher in samples collected from thiopental sodium anaesthetised rats compared to samples collected from conscious decapitated normotensive and 2K1C hypertensive rats (Huskova et al., 2006).

The concentration of Ang (1–7) in the renal cortex of the clipped kidney of 2K1C rats was comparable to that in the renal cortex of the sham group. This might be related to the decreased ACE2 levels in the clipped kidney that were previously reported in 2K1C (Prieto et al., 2011). This along with the increased Ang II levels in the clipped kidney as demonstrated in the present study resulted in minimal changes in Ang (1–7) levels. This could also explain the anti‐diuresis and anti‐natriuresis of the clipped kidney in response to the prevailing levels of Ang II and the limited counteracting effects from endogenous Ang (1–7). The latter influence becomes more prevalent upon the exogenous administration of Ang (1–7) into the clipped kidney in the present study causing increased sodium and water excretion in 2K1C rats. On the other hand, Ang (1–7) concentration was higher in the renal cortex of the non‐clipped kidney of 2K1C rats compared to the corresponding right renal cortex of sham 2K1C rats. Similarly, a significantly higher concentration of Ang (1–7) was revealed in the non‐clipped kidney of conscious decapitated 2K1C mice compared to Ang (1–7) levels in sham kidneys, measured by radioimmunoassay (Rakusan et al., 2010). This finding however is in contrast to that reported by Prieto et al. (2011) using conscious decapitated rats where the non‐clipped kidney, as well as the clipped kidney, had decreased renal tissue levels of Ang (1–7). The reason for these discrepancies is unclear but it is possible that the anaesthesia might affect Ang (1–7) concentrations in plasma and kidney tissue in the present study, which might differentially affect normotensive and 2K1C hypertensive animals, potentially accounting for the differences. Moreover, the length and specifications of the clip application might also be factors in determining the level and duration of ischaemia in the clipped kidney compared to other studies. It is worth noting, however, that the increase in the concentration of Ang (1–7) in the renal cortex of the non‐clipped kidney in the present study was accompanied by minimal changes in tissue concentration of Ang II. This finding was consistent with that mentioned previously regarding the clipped kidney levels of Ang II/Ang (1–7) where a balanced relationship might exist between the two peptides, which essentially determines the renal excretory functional responses in hypertension.

Interestingly, in this study, a significant increase in the levels of Ang (1–7) was revealed in the renal cortex of DOCA‐salt‐treated rats compared to sham DOCA rats. Moreover, renal Ang (1–7) levels were reportedly unchanged in rats fed a high sodium diet (O'Neill et al., 2013), in which blood pressure was similar to rats fed with normal sodium diet. It is possible, in addition to a reduced Ang II production, that an upregulation of ACE2 increases Ang II breakdown into Ang (1–7) (Crackower et al., 2002). However, ACE2 expression was not measured in this study. The later suggestion is less likely as both aldosterone and high salt diets have been found to downregulate ACE2 (Bernardi et al., 2012), unless the combination of DOCA‐salt and salt co‐treatment and reduced renal mass alters the RAS differently from any one of the treatments on their own. Moreover, the mechanism of angiotensin fragment metabolism to form Ang (1–7) involves ACE2‐independent pathways, which were not measured in the present study.

The protein expression of AT_1_R in the renal cortex of the clipped kidney in 2K1C rats compared to sham 2K1C rats, although decreased, did not reach statistical significance. However, the protein expression in the medulla of the clipped kidney of 2K1C group was significantly lower compared to sham 2K1C group. A previous study examined mRNA levels of AT_1_Rs and showed decreased levels in the clipped kidney of 2K1C rats after 2 days of clipping, without any alteration in AT_1_R mRNA in the non‐clipped kidney compared to sham kidneys (Della Bruna et al., 1995). Similarly, AT1a receptor protein expression in both kidneys of 2K1C rats was decreased after 1 week of clipping (Wang et al., 1999). Conversely, Sadjadi et al. (2002) reported no changes in the AT_1_R protein expression in the renal tubules of either the clipped kidney or the non‐clipped kidney of 2K1C rats. Indeed, the relative time‐related change in RAS activity and how it affects the development and maintenance of hypertension in the 2K1C model is not fully established. From the results of this study, it could be deduced that there was decreased AT_1_R protein expression in the clipped kidney of the 2K1C group compared to the sham 2K1C group. The Mas receptor protein expression in the cortex or medulla of the non‐clipped kidney of 2K1C rats was not different from sham 2K1C rats. This is in agreement with a previous study from this lab where Mas receptor protein expression did not change significantly in the renal cortex and medulla of rats fed a low sodium diet (O'Neill et al., 2013). Similarly, a study by Kim et al. (2016) showed that Mas receptor expression in the renal medulla of clipped and non‐clipped kidney 2K1C rats was comparable to sham 2K1C rats. It can therefore be suggested that the altered renal functional responses to Ang (1–7) in the 2K1C rats observed in this study are not likely to be explained by alterations in the protein expression of renal Mas receptors.

This study showed that AT_1_R expression was significantly higher in the renal cortex of DOCA‐salt‐treated rats compared to sham DOCA rats. The upregulation of AT_1_R protein expression in the renal cortex of DOCA‐salt‐treated rats might explain the increased sensitivity to Ang II infusion in this model (Gavras et al., 1975). However, it has been suggested that the increased activation of the AT1a receptor might provide protection against glomerular injury due to hypertension in this model (Hisamichi et al., 2017). The increase in AT_1_R expression in the renal cortex of DOCA‐salt‐treated rats was associated with a downregulation of the Mas receptor in both the renal cortex and the medulla. In addition, the increase in renal cortical expression of AT_1_R and the decrease in Mas receptor expression is in line with the decreased Ang II and increased Ang 1–7 concentrations in the DOCA‐salt‐treated and sham rats. The gene expression pattern for DOCA‐salt hypertension is different from that reported in rats fed a high sodium diet in a previous study from this lab where AT_1_R expression was decreased in the renal cortex of these rats but with no changes in the renal cortical or medullary Mas receptor expression (O'Neill et al., 2013). This suggests that high salt intake, although involved with DOCA in the manifestations of DOCA hypertensive rat model, constitutes a disease model which could regulate RAS peptide levels differentially from DOCA treatment itself. Together, the altered balance of angiotensin fragments to favour Ang (1–7) in addition to the altered expression of angiotensin receptors to favour AT_1_Rs could likely contribute to the differential Ang (1–7) signalling in response to the intrarenal infusion of exogenous Ang (1–7). This notion is supported by studies showing that Ang (1–7) infusion in DOCA‐salt‐treated rats did not halt the progression of hypertension or cardiac hypertrophy (Grobe et al., 2006). However, the combined treatment with a Mas receptor agonist and a renin inhibitor had a cumulative blood pressure lowering effect in the DOCA model (Singh et al., 2013).

In conclusion, the intrarenal infusion of Ang (1–7) into the clipped kidney of 2K1C rats elicited diuretic and natriuretic responses with no change in GFR. This appeared to be linked to the degree of RAS activation in the clipped kidney. Intrarenal Ang (1–7) infusion into the non‐clipped kidney of 2K1C rats caused sodium and water retention, potentially through AT_1_R signalling. In support of this, the intrarenal infusion of Ang (1–7) caused sodium and water retention in DOCA‐salt‐treated hypertensive rats, where Ang II levels and AT_1_R expression were decreased. Rather than simply being dependent on endogenous RAS activation, the findings suggest that the renal actions of Ang (1–7) are dependent on the relative endogenous levels of each arm of the classical Ang II–AT_1_R axis with those of the Ang (1–7)*–*Mas receptor axis. Therefore, in states of increased or unchanged endogenous levels of Ang (1–7) but normal or increased Ang II, exogenous Ang (1–7) has natriuretic actions. By contrast, in states of unchanged or decreased endogenous Ang II and elevated Ang (1–7), exogenous Ang (1–7) exerts an anti‐natriuretic action. Thus, the relative balance between the intrarenal classical and novel arms of the RAS, and in particular the relative abundance of AT_1_R to Mas receptor may to a large extent determine the renal excretory response to Ang (1–7) infusion. Further studies are necessary to examine the effects of exogenously administered Ang (1–7) on the renal functional parameters in female 2K1C and DOCA‐salt hypertensive rats. Previous studies have shown that Ang (1–7) contributes to the differential blood pressure responses to Ang II in female compared to male spontaneously hypertensive rats (Sullivan et al., 2010). In this regard, female rats were found to have higher renal cortical Ang (1–7) levels than male rats and were less sensitive to the hypertensive effects of Ang II compared to male rats. However, both female and male spontaneously hypertensive rats had similar renal cortical AT1 and Mas receptor expression in the aforementioned study. Moreover, Lee et al. (2019) showed that there is high expression of intratubular Ang (1–7) and Mas receptors in the clipped kidney of female 2K1C rats relative to male 2K1C rats. It could therefore be hypothesised that female 2K1C and DOCA‐salt rats would exhibit altered renal functional responses to exogenously administered Ang (1–7) compared to male rats, and this warrants further investigation.

## AUTHOR CONTRIBUTIONS

Elaine F. Barry and Edward J. Johns conceived and designed the experiments for this study. Elaine F. Barry performed the experiments and collected the data. Mohammed H. Abdulla, Sara AlMarabeh, Julie O'Neill, Julie Beshara and Erin Parna‐Gile interpreted the data and drafted the manuscript. All authors have read and approved the final version of this manuscript and agree to be accountable for all aspects of the work in ensuring that questions related to the accuracy or integrity of any part of the work are appropriately investigated and resolved. All persons designated as authors qualify for authorship, and all those who qualify for authorship are listed.

## COMPETING INTERESTS

The authors declared no potential conflicts of interest with respect to the research, authorship and/or publication of this article.

## Supporting information

Statistical Summary Document

## Data Availability

The data that support the findings of this study are available on request from the corresponding author.
